# Increased nitrate intake from beetroot juice over 4 weeks affects nitrate metabolism, but not vascular function or blood pressure in older adults with hypertension†

**DOI:** 10.1039/d3fo03749e

**Published:** 2024-03-25

**Authors:** Rebeka Fejes, Martin Lutnik, Stefan Weisshaar, Nina Pilat, Karl-Heinz Wagner, Hans-Peter Stüger, Jonathan M. Peake, Richard J. Woodman, Kevin D. Croft, Catherine P. Bondonno, Jonathan M. Hodgson, Michael Wolzt, Oliver Neubauer

**Affiliations:** a Department of Nutritional Sciences, Research Platform Active Ageing, University of Vienna Vienna Austria oliver.neubauer@univie.ac.at; b Vienna Doctoral School of Pharmaceutical, Nutritional and Sport Sciences, University of Vienna Vienna Austria; c Department of Clinical Pharmacology, Medical University of Vienna Vienna Austria; d Department of Cardiac Surgery, Medical University of Vienna Vienna Austria; e Division Integrative Risk Assessment, Data and Statistics, Austrian Agency for Health and Food Safety Vienna Austria; f School of Biomedical Sciences, Queensland University of Technology Queensland Australia; g Flinders Centre for Epidemiology and Biostatistics, Flinders University Adelaide South Australia Australia; h Medical School, University of Western Australia, Royal Perth Hospital Unit Perth Western Australia Australia; i Nutrition & Health Innovation Research Institute, School of Medical and Health Sciences, Royal Perth Hospital Research Foundation, Edith Cowan University Joondalup Western Australia Australia; j Centre for Health Sciences and Medicine, University for Continuing Education Krems Krems Austria

## Abstract

The decline in vascular function and increase in blood pressure with aging contribute to an increased cardiovascular disease risk. In this randomized placebo-controlled crossover study, we evaluated whether previously reported cardiovascular benefits of plant-derived inorganic nitrate *via* nitric oxide (NO) translate into improved vascular function and blood pressure-lowering in 15 men and women (age range: 56–71 years) with treated hypertension. We investigated the effects of a single ∼400 mg-dose at 3 hours post-ingestion (3H POST) and the daily consumption of 2 × ∼400 mg of nitrate through nitrate-rich compared with nitrate-depleted (placebo) beetroot juice over 4 weeks (4WK POST). Measurements included nitrate and nitrite in plasma and saliva; endothelial-dependent and -independent forearm blood flow (FBF) responses to acetylcholine (FBF_ACh_) and glyceryltrinitrate (FBF_GTN_); and clinic-, home- and 24-hour ambulatory blood pressure. Compared to placebo, plasma and salivary nitrate and nitrite increased at 3H and 4WK POST following nitrate treatment (*P* < 0.01), suggesting a functioning nitrate-nitrite-NO pathway in the participants of this study. There were no differences between treatments in FBF_ACh_ and FBF_GTN_-area under the curve (AUC) ratios [AUC ratios after (3H POST, 4WK POST) compared with before (PRE) the intervention], or 24-hour ambulatory blood pressure or home blood pressure measures (*P* > 0.05). These findings do not support the hypothesis that an increased intake of dietary nitrate exerts sustained beneficial effects on FBF or blood pressure in hypertensive older adults, providing important information on the efficacy of nitrate-based interventions for healthy vascular aging. This study was registered under ClinicialTrials.gov (NCT04584372).

## Abstract 2

In this randomized placebo-controlled crossover study, we evaluated whether previously reported cardiovascular benefits of plant-derived inorganic nitrate *via* NO translate into improved vascular function and blood pressure-lowering in middle-aged and older men and women with treated hypertension.

## Graphical abstract

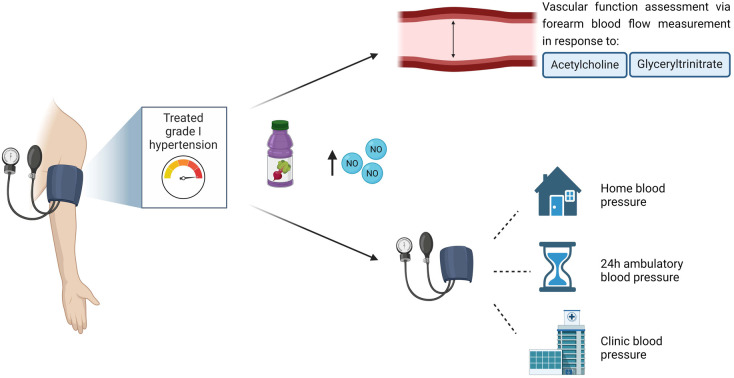

## Introduction

Cardiovascular diseases (CVD) remain the leading cause of morbidity, disability, and mortality in modern societies.^1,2^ The risk of CVD increases with aging.^1,3^ Age-associated vascular dysfunction and high blood pressure (or hypertension) are main risk factors for CVD.^2,4^ Developing effective lifestyle interventions to promote, preserve or restore vascular function, and to prevent or treat hypertension with advancing age is a high priority for biomedical research.^5^ The diet composition has an important influence on cardiovascular health.^6^ A strong and consistent body of evidence from observational studies has shown that dietary patterns rich in vegetables are associated with a reduced CVD risk.^6,7^ Emerging data from observational and experimental studies suggest that inorganic nitrate is a key bioactive compound of vegetables in this regard.^8–10^ Dietary nitrate, found abundantly in green leafy vegetables and some root vegetables, is an important source of nitric oxide (NO), a signaling molecule that is central for cardiovascular function.^10–12^ Endogenously, NO is produced by nitric oxidases (NOS), including endothelial NOS (eNOS), but the vascular availability of NO is reduced during aging due to oxidative stress, low-grade inflammation, an enhanced NO degradation, and a decreased NO production by eNOS.^9–11^ The decreased NO bioavailability is associated with impaired vascular endothelial function, vascular aging, and an increased CVD risk.^3,12,13^ A growing and compelling body of evidence indicates that achievable increases in the nitrate intake from plant foods exert beneficial NO-mediated physiological effects on the cardiovascular system.^8,9,11^ Previous intervention studies from our group and others have reported favorable^14–19^ or no effects^20–22^ of nitrate, most commonly from beetroot juice or leafy green vegetables, on blood pressure^16–19,21,22^ and endothelial function^14–18,21,22^ in middle-aged and older adults with^15,16,18,20,22^ or without^14,17,19,21^ CVD risk factors. In hypertensive individuals on anti-hypertensive medication, the efficacy of nitrate to improve blood pressure and vascular function is apparently dependent on the degree of blood pressure elevation and vascular dysfunction and not anti-hypertensive medication status.^16,18^

Importantly, however, available information on whether an increased nitrate intake over ≥4 weeks affects cardiovascular health-related outcomes in older populations and populations with an elevated risk for CVD is still incomplete.^8^ There is therefore a strong need to investigate the impact of chronic nitrate intake on cardiovascular health in these population groups. Furthermore, in only a few studies, both acute (≤24 hours) effects following the ingestion of a single nitrate dose and longer-term effects after regular consumption of nitrate from the same dietary source have been investigated.^15^ This is noteworthy, considering that different physiological mechanisms might be involved in the acute as compared with the chronic cardiovascular effects of nitrate.^9,11^ Another important aspect is that in previous human studies, endothelial function was mostly assessed by measuring brachial artery flow-mediated dilatation (FMD).^8,23^ This method provides a measure of macrovascular (conduit artery) function,^3,5^ but it does not offer insights into microvascular (resistance artery) function. This is worth noting, because the onset of the decline in microvascular function might precede the decline in macrovascular function.^24,25^

To gain a better understanding on the effects of dietary nitrate on resistance artery function, as reported herein, we applied strain gauge plethysmography and measured forearm blood flow (FBF) in response to the infusion of vasoactive substances into a brachial artery, as an accurate, reproducible, and commonly used method for assessing microvascular function.^5,13,14,26^ In particular, FBF measurement following infusion of the endothelium-dependent vasodilator acetylcholine (ACh), an agonist for NO production through eNOS (FBF_ACh_), is a clinically relevant measure of vascular endothelial function.^5,27,28^ FBF_ACh_ is reduced with aging^5^ and independently predictive of future cardiovascular events and mortality in men and women.^3,5,29^ In addition, measuring ‘endothelium-independent’ responses of FBF in response to infusion of the NO donor glyceryltrinitrate (GTN) (FBF_GTN_) provides a measure of the vasodilatory responsiveness of vascular smooth muscle cells in the arterial wall to NO.^5,28^ Age-related changes in the vascular smooth muscle tone and structural components of the arterial wall may contribute to artery stiffness and endothelial dysfunction.^30^

The primary aim of this randomized, double-blind, placebo-controlled crossover study was to test the hypothesis whether an increased intake of dietary nitrate over 4 weeks improves endothelial-dependent dilatation, as assessed by measuring FBF_ACh_, and lowers 24-hour ambulatory blood pressure in middle-aged and older men and women with treated hypertension. We also investigated the effects of nitrate-rich *versus* nitrate-depleted beetroot juice following the intake of a single dose (acute) and the regular consumption over 4 weeks (chronic) on endothelial-dependent and -independent mechanisms underlying vascular function, as assessed by FBF_ACh_, and FBF_GTN_, and blood pressure.

## Materials and methods

### Study participants

Fifteen ambulant men and women, between the ages of 56 and 71 years, who had been diagnosed with hypertension and who were taking anti-hypertensive medication, completed this study. The study was approved by the Ethics Committee of the Medical University of Vienna (ethics number: EK 2238/2018) and is publicly registered under ClinicalTrials.gov (NCT04584372). All participants provided written, informed consent before their enrolment into the study. Prior to the enrolment, participants completed an examination by a medical physician. This examination included collection of information about medical history and current medications, resting electrocardiography (ECG), anthropometric and blood pressure measurements, and a routine laboratory analysis of fasting blood and urine samples. Furthermore, they completed validated physical activity-^31^ and food frequency-questionnaires^32^ to obtain their physical activity level and baseline dietary intake. For inclusion into the study, participants had to have a resting mean systolic blood pressure between 130 and 170 mmHg, inclusive. Participants were excluded if there was any evidence of acute or chronic disease such as symptomatic cardiovascular or peripheral vascular disease, and if they had a systolic blood pressure of <130 or >170 mm Hg or a diastolic blood pressure of >110 mmHg, a fasting glucose of >7.0 mmol L^−1^, or a body mass index (BMI, in kg m^−2^) of <18.5 or >35. Additional exclusion criteria were consumption of ≥5 serves of vegetables per day; consumption of a diet estimated to contain >200 mg nitrate per day; being vegan or vegetarian; chronic use of nitric oxide (NO) donors, organic nitrites/nitrates, sildenafil and related drugs, non-steroidal anti-inflammatory or statin-related drugs; use of antibacterial mouthwash (to avoid interference of the mouthwash with nitrate-reducing oral bacteria^33^); use of antibiotics (within previous 2 months); a change in drug therapy likely to influence blood pressure or major secondary outcomes within the previous month, or the likelihood that drug therapy would change during the study; current or previous engagement in planned, structured, and regular exercise training involving more than two hours of net exercise time per week; current or recent (<6 months) loss or gain of >6% of body weight; current or recent (<12 months) regular smoking; and alcohol intake of >140 g per week for women or >210 g per week for men and/or binge drinking behavior.

### Study overview

**Fig. 1:**
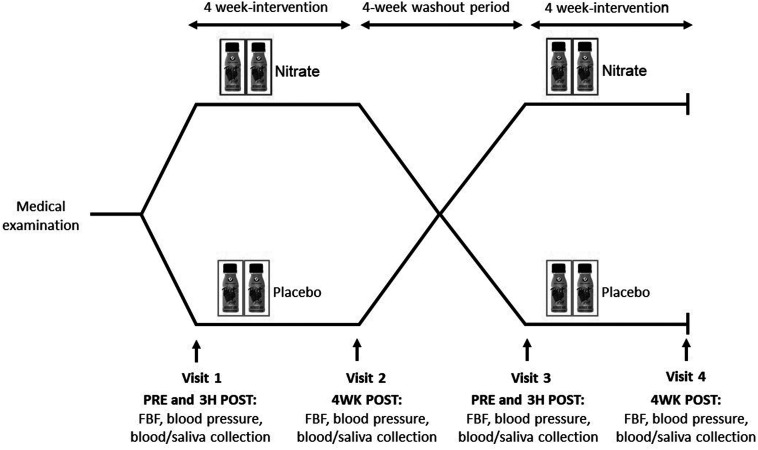
Study design. FBF, forearm blood flow.

The study involved a randomized, double-blind, placebo-controlled crossover design, with two 4-week treatment periods separated by a 4-week washout period. The two treatments consisted of a 4-week intervention with nitrate-rich beetroot juice and a 4-week intervention with nitrate-depleted beetroot juice (placebo), on the background of a low-nitrate diet and unaltered lifestyle (described below). The pre-defined primary endpoints were the FBF_ACh_-area under the curve (AUC) ratio after compared with before the 4-week (nitrate *versus* placebo) treatment periods, and 24-hour ambulatory systolic blood pressure before and after the 4-week (nitrate *versus* placebo) treatment periods. Post- *versus* pre-intervention FBF-AUC ratios are widely recognized as the most robust outcome measures of bilateral strain gauge plethysmography.^26,28^ Monitoring 24-hour blood pressure is the preferred diagnostic method for assessing (high) blood pressure.^4^ The study design is summarized in Fig. 1.

After the medical examination confirmed eligibility, the participants were enrolled into the study and allocated to one of the two study treatments *via* a computer-generated block random assignment. Participants and all investigators involved in this study were blind to the treatment allocation. For the background diet, the participants were instructed to maintain all meals as usual except for minimizing the intake of nitrate-rich foods. A list of foods to avoid or limit was provided. They were asked to maintain other lifestyle factors and medication throughout the study period. Adherence to the study protocol was verified by using standard questionnaires, including physical activity-^31^ and (modified) food frequency-questionnaires,^32^ at each of these visits. In addition to the screening visit, each participant completed a total of 4 study visits for measurements of resting blood pressure and FBF and collection of blood and saliva samples at the Department of Clinical Pharmacology of the Medical University of Vienna, located at the Vienna General Hospital.

To examine the clinical outcome and any chronic effects of the 4-week nitrate *versus* placebo treatments, the study visits were scheduled on the first day (PRE) and in the morning after the last day (4WK POST) of each of the intervention periods. For all study visits, the participants were required to arrive at the research clinic between 08 : 00 and 09 : 00 a.m. in a fasted state following an overnight fast. In the 24 hours preceding each of the visits, they were required to refrain from caffeine and alcohol consumption and to avoid any strenuous exercise. Prior to the study visits 4 weeks post-treatment, participants consumed their final nitrate-rich or nitrate-depleted beetroot juice ≥12 hours before these visits.

To examine any acute effects of nitrate-rich and nitrate-depleted beetroot juice on the first day of each treatment, the participants consumed their first dose (70 mL) of the allocated nitrate-rich or nitrate-depleted beetroot juice on the first day of each of the intervention periods at the clinic. They then consumed a standardized, low-nitrate breakfast (80 g high-fiber low-sugar cereal biscuits, 200 mL milk), as in our previous study.^19^ In addition to the resting measurements and sample collections in the fasted state, resting blood pressure and FBF were measured, and blood and saliva were collected 3 hours after beetroot juice consumption (3H POST).

### Dietary intervention

The two treatments were as follows: (1) daily consumption of 2 × 70 mL nitrate-rich beetroot juice (70 mL with breakfast and 70 mL with dinner); (2) daily consumption of 2 × 70 mL nitrate-depleted beetroot-juice as placebo (70 mL with breakfast and 70 mL with dinner), both for a treatment period of 4 weeks. All products were obtained from the same supplier (Beet It, James White Drinks Ltd., UK). According to the manufacturer batch analysis, 70 mL of nitrate-rich beetroot juice contained 6.45 mmol (∼400 mg) nitrate, whereas 70 mL of nitrate-depleted beetroot juice contained ≤0.02 mmol nitrate. The daily doses over the 4-week long treatment periods were 12.9 mmol (∼800 mg) nitrate from 2 × 70 mL of nitrate-rich beetroot juice, and ≤0.04 mmol nitrate from 2 × 70 mL of nitrate-depleted beetroot juice.

### Blood plasma and salivary nitrate and nitrite analysis

At each measuring time-point, blood was collected through a 21-gauge butterfly needle inserted into an antecubital vein. Blood was collected into vacutainers (Greiner Bio-One GmbH, Austria), containing lithium-heparin. For plasma analyses, blood samples were centrifuged at 3500 rpm for 15 min at 4 °C. Saliva was collected through Salivette® tubes (Sarstaed Ges.m.b.H., Austria), and centrifuged at 1000*g* for 2 min at 20 °C. Plasma and saliva were aliquoted and stored at minus 80 °C until analysis.

Nitrate and nitrite concentrations were measured in plasma and saliva, using gas-chromatography-mass spectrometry with ^15^N-labeled nitrate and nitrite as internal standards, as previously described.^34^ In addition, the nitrate content of the nitrate-rich and nitrate-depleted (placebo) beetroot juice beverages were measured in our laboratory to confirm the accuracy of the manufacturer's analysis, using the same method.

### Forearm blood flow (FBF) measurements

Forearm blood flow (FBF) was assessed in both arms by strain gauge plethysmography, as described previously.^28^ Briefly, strain gauges, placed on both forearms, were connected to plethysmographs (EC-6; D.E. Hokanson Inc., Bellevue, WA, USA) for measurement of changes in forearm volume in response to inflation of congesting cuffs on the upper arms to a supravenous pressure (45 mmHg). The distances from the elbows to the strain gauges were measured to ensure a comparable setting between study days. Measurements were recorded for 9 seconds at 30-second intervals during repeated inflation of the upper arm cuffs. The early linear increases of the curves were used for FBF analysis using the NIVP3 software (version 5.27, D.E. Hokanson Inc., Bellevue, WA, USA) and are expressed as ml min^−1^ 100 mL^−1^ forearm volume forearm volume. All FBF measurements at each measuring-timepoint (*i.e.*, PRE, 3H POST, 4WK POST) during the nitrate and placebo treatment periods were performed in a quiet room with an ambient constant temperature of 23 °C (±1 °C). Participants were in a supine position and a 27 gauge fine-bore needle (Sol Care, Sol-Millennium Medical Inc., Lawrenceville, GA, USA) was inserted into the brachial artery of the non-dominant arm for intra-arterial administration of vasoactive substances. Control 0.9% sodium chloride solution (Fresenius Kabi, Graz, Austria) FBF measurements were recorded over 5 minutes followed by assessing the response to the endothelium-dependent vasodilator ACh (Miochol-E®, Bausch & Lomb Swiss AG, Switzerland) at increasing doses of 25, 50 and 100 nmol min^−1^ (each for 3 minutes), respectively. To test smooth muscle function, the endothelium-independent dilator GTN (Nitro POHL, G. Pohl-Boskamp GmbH & Co. KG, Germany; 4, 8 and 16 nmol min^−1^; each for 3 min) was administered after a 15 minutes-washout period to allow restoration of control FBF conditions.

### Blood pressure measurements

#### Clinic blood pressure

Clinic blood pressure was measured with an automatic device (Infinity Delta monitoring system; Drägerwerk AG&Co, KGaA, Germany). Three measurements were performed after ≥10 min of rest with two-minute breaks in between in a quiet room in the clinic with an ambient constant temperature of 23° C (±1° C). The mean of these three measurements was used for analysis.

#### Home blood pressure

Participants were provided with a digital blood pressure monitor (Boso medicus X, Bosch&Sohn, Germany) and an appropriately sized upper arm cuff, and were given appropriate instructions on its use before they commenced the study. Home blood pressure was measured and recorded by each participant 3 times daily (shortly after waking and before breakfast, 1–2 hours before dinner, and 1–2 hours after dinner) for the entire study duration. Participants conducted three blood pressure readings at each of the 3 daily measuring time-points taking two-minute breaks in between. The first measurement was omitted from the analysis, and the mean of the second and third measurements was used for data analysis. Mean blood pressure was assessed for each of the entire 4 weeks long intervention periods.

#### 24-hour ambulatory blood pressure

Ambulatory blood pressure and heart rate were monitored for a 24-hour period prior to all study visits (*i.e.*, prior to and at the end of the nitrate and placebo treatments), as described previously.^20^ Recording was performed at 20 min intervals during the day and 30 min intervals during the night, by using a 24-hour blood pressure monitor (Boso TM-2430 PC 2, Bosch&Sohn, Germany). Participants were given appropriate training and instructions on the use of the monitors.

### Statistical analysis

A sample size of *n* = 29 participants completing the study was calculated based on the primary endpoints of FBF_ACh_ for providing 80% power to detect a relative 20% difference in the mean FBF_Ach_ AUC ratio (post- *versus* pre-intervention) and differences of ≥2 mmHg in the mean 24-hour ambulatory systolic blood pressure with an α of 0.05, on the basis of data from previous studies.^20,28^

With regards to the FBF data analysis, as described previously,^28^ FBF was calculated as ml min^−1^ 100 mL^−1^ forearm volume, percent changes, AUC, and AUC ratios. The effects of ACh or GTN (*i.e.*, mean of six measurements at each ACh or GTN dose level) on FBF are expressed as percent changes of ratios of the interventional (*i.e.*, ACh or GTN-infused) arm *versus* the control arm, with individual basal FBF ratios (*i.e.*, the mean of 10 measurements during saline infusion) defined as 100%. The FBF-AUCs were derived from the dose-effect curves according to the linear trapezoidal rule restricted to the range of active dosing consisting of three data points (*i.e.*, 25, 50, 100 nmol min^−1^ for ACh, and 4, 8, 16 nmol min^−1^ for GTN, respectively).

Statistical analyses were performed using R Studio (RStudio 2022.12.0 + 353 “Elsbeth Geranium” release for Windows) and Microsoft Excel (2019 MSO 64-Bit). The gathered data was organized and checked for completeness, accuracy, and missing values. A repeated-measures linear mixed-model was used to compare differences between treatments (nitrate *versus* placebo) adjusted for baseline for each outcome variable, and for investigating within treatment effects between baseline (PRE) *versus* acute (3H POST) and chronic (4WK POST) values, respectively. For the pre-defined primary outcomes (*i.e.*, FBF_ACh_-AUC ratio after compared with before the 4-week nitrate *versus* placebo interventions, 24-hour ambulatory blood pressure), the Tukey *post hoc*-correction was applied to account for multiple comparisons. The sequence of the given treatments was included as a fixed effect in the model to capture any potential carryover effects resulting from the crossover design. Statistical significance was set at a *P*-value of <0.05. Data are presented as mean ± standard deviation (SD), unless stated otherwise.

## Results

### Study participants

**Fig. 2:**
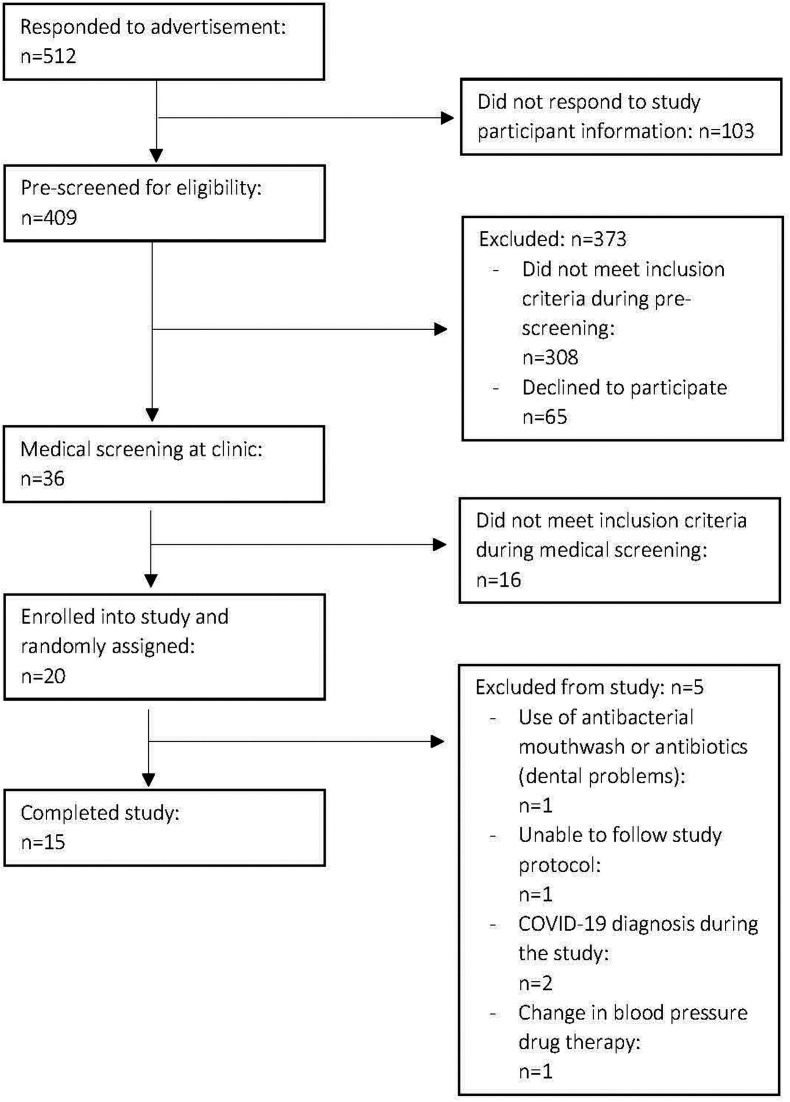
Consort flow diagram for participant recruitment. Consort, consolidated standards of reporting trials.

The study started on January 1^st^, 2021, and final data were collected on March 28^th^, 2023. Of the 409 participants pre-screened for the study, 389 volunteers were excluded during the pre-screening and the medical screening at the clinic (mainly due to use of medications specified as exclusion criteria), 20 were randomly assigned, 15 of whom completed the study. Five participants dropped out from the study after randomization due to reasons unrelated to the study. Details about the participant recruitment are summarized in Fig. 2. Table 1 shows baseline anthropometric and clinical characteristics for the 15 participants completing the study.

**Table 1:** Baseline anthropometric and clinical characteristics and medications of the study participants

Anthropometric and clinical characteristics	Baseline values
Age (years)	62.5 ± 4.8
Sex (male : female)	10 men, 5 women
Body mass index (kg m^−2^)	26.5 ± 3.7
Baseline systolic blood pressure (mmHg)	146.1 ± 10.1
Baseline diastolic blood pressure (mmHg)	90.0 ± 8.8
Baseline mean arterial pressure (mmHg)	108.7 ± 8.0
Baseline pulse (bpm)	67.1 ± 7.8
Waist circumference (cm)	97.2 ± 14.3
Hip circumference (cm)	105 ± 6.0
Medications	
Angiotensin receptor blockers	10
Calcium channel blockers	7
Beta blockers	3
Alpha-1 blockers	2
Platelet aggregation inhibitors	1
Diuretics	2
Angiotensin-converting-enzyme inhibitors	2
Thyroid medications	2
Proton-pump inhibitors	1

### Blood plasma and salivary nitrate and nitrite

**Fig. 3:**
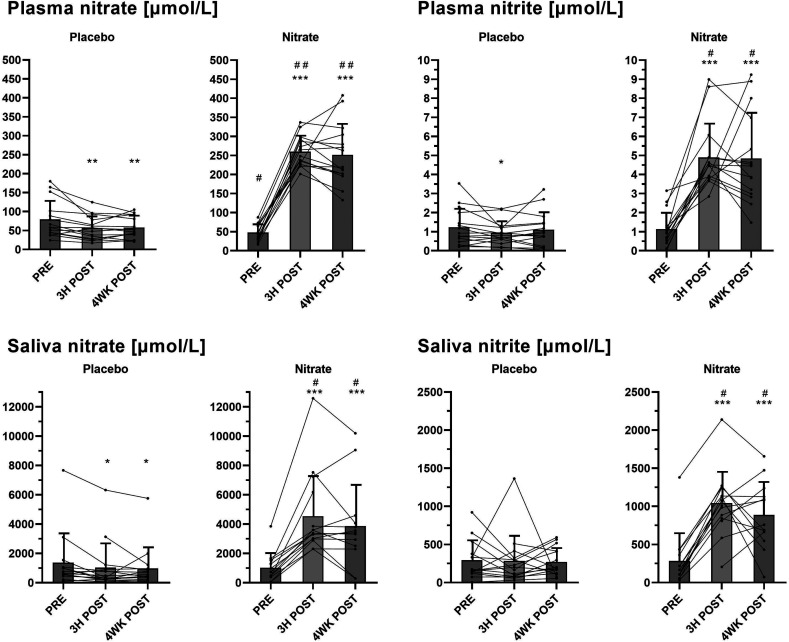
Plasma and salivary concentrations of nitrate and nitrite before (PRE), and 3 hours (3h post) after intake of the first dose of nitrate-rich (nitrate; ∼400 mg nitrate) *versus* nitrate-depleted (placebo) beetroot juice, and 4 weeks (4WK POST) after daily nitrate-rich (nitrate; ∼800 mg nitrate) *versus* nitrate-depleted (placebo) juice consumption. Group data (bars) are presented as mean ± sd, and individual data are indicated with circles (*n* = 15; 10 men, 5 women). PRE *versus* POST: *, *p* < 0.05; **, *p* < 0.01; ***, *p* < 0.001; nitrate *versus* placebo: #, *p* < 0.01; ##, *p* < 0.001. Data were compared using a repeated-measures linear mixed-model.

Plasma and salivary concentrations of nitrate and nitrite at baseline (PRE), 3H POST and 4WK POST following treatments with nitrate-rich and nitrate-depleted beetroot juice are shown in Fig. 3. Significant (∼4- to 5-fold) increases in plasma concentrations of both nitrate and nitrite were observed at 3H POST and 4WK POST following nitrate-rich beetroot juice treatment, but not the placebo treatment, and plasma nitrate and nitrite levels were significantly different between the treatments (*P* < 0.001). Salivary nitrate and nitrite concentrations increased (∼3- to 4-fold) 3H POST and 4WK POST following the nitrate treatment only, with significant differences between the nitrate and placebo treatments (*P* < 0.001 for both timepoints).

### Forearm blood flow (FBF) measurements

**Fig. 4:**
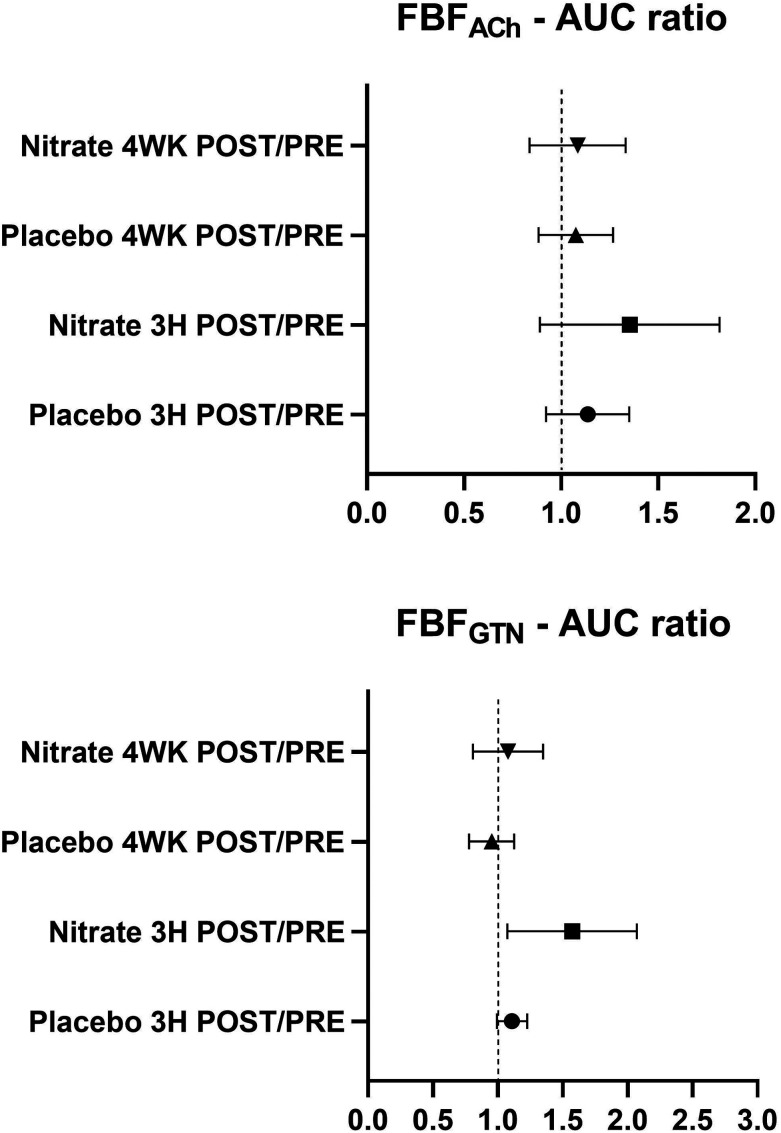
POST- *versus* pre-intervention forearm blood flow (FBF)-area under the curve (AUC) ratios for FBF measurements in response to acetylcholine (ACH) or glyceryltrinitrate (GTN) 3 hours (3H POST) *versus* baseline (PRE) and 4 weeks (4WK POST) *versus* PRE after the interventions with nitrate-rich (nitrate) or nitrate-depleted (placebo) beetroot juice. Data are presented as mean and 95% confidence interval (*n* = 15; 10 men, 5 women). Data were compared using a repeated-measures linear mixed-model and a tukey *post hoc*-correction.

**Fig. 5:**
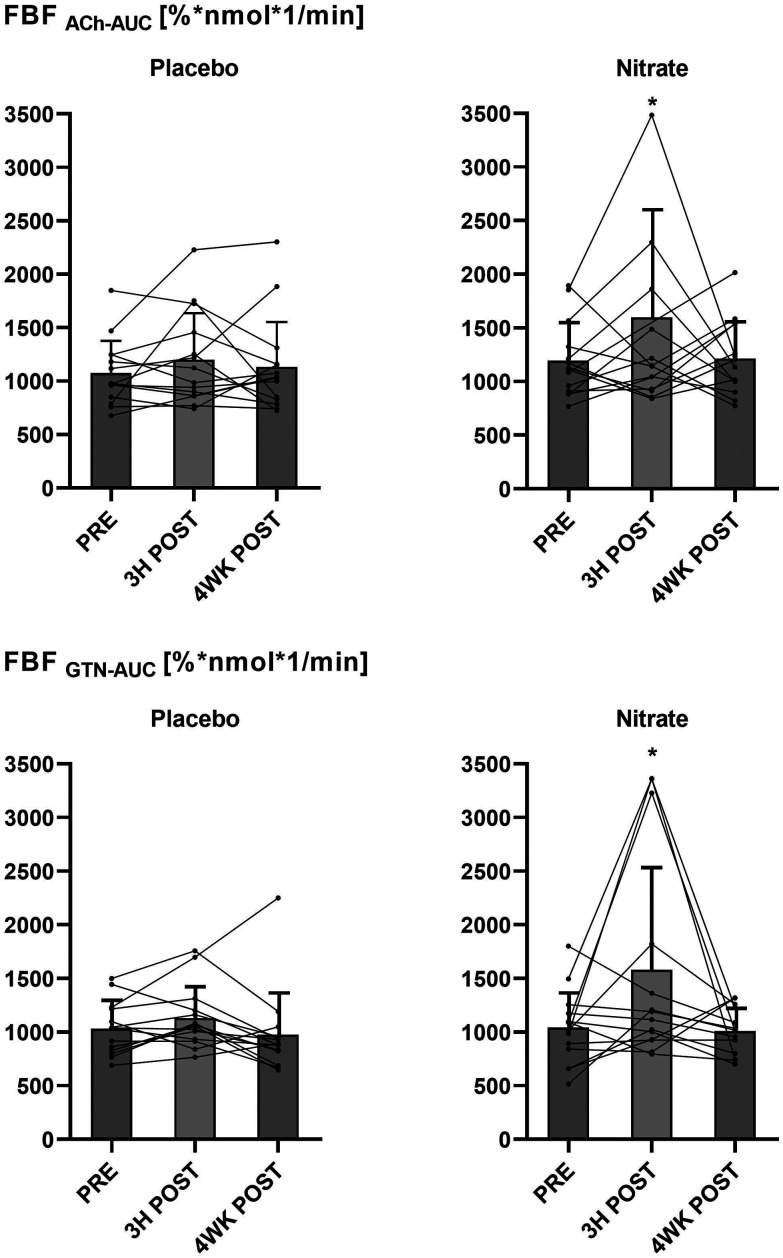
Forearm blood flow (FBF) responses calculated as area under the curve (AUC) in the interventional- *versus* control arm in response to acetylcholine (FBF_ACh_-AUC) and glyceryltrinitrate (FBF_GTN_-AUC) before (PRE), and 3 hours (3H POST) and 4 weeks (4WK POST) after the interventions with nitrate-rich (nitrate) or nitrate-depleted (placebo) beetroot juice. Group data (bars) are presented as mean ± sd, and individual data are indicated with circles (*n* = 15; 10 men, 5 women). PRE *versus* 3H POST: *, *p* < 0.05. Data were compared using a repeated-measures linear mixed-model.

There were no differences in basal FBF between the interventional- and the control arm during intra-arterial saline infusion (ESI Table 1†). The outcome of the nitrate *versus* the placebo treatments on FBF responses to intra-arterial ACh (FBF_ACh_) and GTN (FBF_GTN_), analyzed and expressed as AUC ratios after (3H POST, 4WK POST) compared with before (PRE) the treatments is shown in Fig. 4. Post- *versus* pre-intervention FBF ratios were not different between the nitrate and placebo treatments. The FBF responses calculated as interventional- *versus* control arm AUC in response to ACh (FBF_ACh_-AUC) and GTN (FBF_GTN_-AUC) before (PRE) and after (3H POST, 4WK POST) the nitrate *versus* placebo treatments are summarized in Fig. 5. There were no differences between treatments arms. Compared to baseline, FBF_ACh_-AUC and FBF_GTN_-AUC increased at 3H POST following the nitrate treatment (for both *P* < 0.05), but not following the placebo treatment. ESI Fig. 1† presents the percentage changes of vasodilation of the interventional (*i.e.*, ACh or GTN-infused) arm *versus* the control arm (FBF ratio [%]) at different (ACh and GTN) dose levels at PRE, 3H POST, and 4WK POST during the nitrate *versus* placebo treatment periods.

### Blood pressure measurements

The mean ± SD measures for clinic, home-, and 24-hour ambulatory blood pressure at baseline and following the nitrate-rich and nitrate-depleted beetroot juice treatments are presented in Table 2. Systolic blood (SBP) and diastolic blood (DBP) pressure measures were used to calculate mean arterial pressure (MAP = DBP + [(SBP – DBP) × 0.33]). There were no differences between the nitrate-rich and nitrate-depleted treatments in home blood pressure or in 24-hour ambulatory blood pressure measures. Relative to baseline, a decrease in systolic and mean arterial clinic blood pressure was observed at 3H POST following treatment with nitrate-rich beetroot juice (*P* < 0.05), and at 4WK POST after treatment with nitrate-depleted beetroot juice (*P* < 0.05).

**Table 2:** Clinic, home, and 24-hour ambulatory blood pressure measurements

		Nitrate					Placebo					Differences between treatments	
		PRE	3H POST	4WK POST	PRE *vs.* 3H POST (*P*-value)	PRE *vs.* 4WK POST (*P*-value)	PRE	3H POST	4WK POST	PRE *vs.* 3H POST (*P*-value)	PRE *vs.* 4WK POST (*P*-value)	3H POST (*P*-value)	4WK POST (*P*-value)
Clinic blood pressure	SYS	137 ± 12	131 ± 9*	135 ± 11	0.029	0.451	140 ± 14	136 ± 16	133 ± 11*	0.249	0.030	0.519	0.130
	DIA	82 ± 9^#^	79 ± 7	82 ± 8	0.088	0.858	85 ± 8	82 ± 12	81 ± 9^#^	0.088	0.059	0.807	0.030
	MAP	100 ± 9	96 ± 7*	100 ± 8	0.043	0.813	103 ± 9	100 ± 12	98 ± 9*^#^	0.120	0.035	0.893	0.042
	PUL	65 ± 10	63 ± 8	66 ± 7	0.251	0.302	67 ± 9	61 ± 6	65 ± 8	<0.001	0.165	0.064	0.091
Home blood pressure	SYS	—	—	135 ± 12	—	—	—	—	135 ± 11	—	—	—	0.652
	DIA	—	—	84 ± 6	—	—	—	—	85 ± 7	—	—	—	0.157
	MAP	—	—	101 ± 8	—	—	—	—	102 ± 7	—	—	—	0.334
	PUL	—	—	67 ± 8	—	—	—	—	68 ± 7	—	—	—	0.310
24-hour ABPM	SYS	129 ± 11	—	131 ± 10	—	0.449	130 ± 13	—	127 ± 10	—	0.151	—	0.123
	DIA	81 ± 8	—	82 ± 6	—	0.468	81 ± 8	—	80 ± 8	—	0.166	—	0.136
	MAP	97 ± 8	—	98 ± 6	—	0.435	97 ± 9	—	96 ± 8	—	0.153	—	0.117
	PUL	68 ± 7	—	70 ± 7*	—	0.045	68 ± 7	—	69 ± 7	—	0.585	—	0.179

## Discussion

The primary finding of this randomized clinical study was that the daily consumption ∼800 mg of inorganic nitrate in the form of 2 × 70 mL nitrate-rich beetroot juice, as compared with nitrate-depleted beetroot juice (placebo), over a period of 4 weeks did not improve vascular function or lower 24-hour ambulatory or home blood pressure in middle-aged and older adults with treated hypertension. By measuring forearm blood flow (FBF) in response to acetylcholine (FBF_ACh_) and glyceryltrinitrate (FBF_GTN_), we were able to assess whether nitrate affects vasodilatory responses of peripheral resistance arteries through endothelial-dependent and endothelial-independent mechanisms. While post- *versus* pre-intervention ratios in FBF_ACh_ (as the primary outcome measure regarding FBF) and FBF_GTN_ were not different between treatments, the intake of a single dose of 70 mL beetroot juice containing ∼400 mg of nitrate increased FBF-AUCs (as secondary FBF outcome measures) three hours following the intake, compared with baseline. Whether the latter suggests that nitrate exerts subtle acute benefits on vascular function needs to be verified in future investigations. From a practical perspective, our study findings do not support our hypothesis that an increased intake of inorganic nitrate from beetroot juice sustainably improves vascular function and lowers blood pressure in this population group.

This offers new information on the efficacy of nitrate-based interventions for healthy vascular aging and adds important knowledge to an active area of research on the physiological and clinical effects of food components.

### Biomarkers of nitrate intake and metabolism indicate functioning nitrate-nitrite-NO pathway

Dietary nitrate exerts its physiological effects through stimulation of the entero-salivary nitrate-nitrite-NO pathway.^8,11,35^ In this pathway, NO is generated following the intake of nitrate contained in the diet (mainly vegetables) involving nitrate-to-nitrite reduction by commensal bacteria in the mouth and further reduction of nitrite to NO by enzymatic and non-enzymatic mechanisms in the blood and tissues.^8,11,35^ In the present study, plasma and salivary nitrate concentrations increased 3 hours after ingestion of the first 70 mL of nitrate-rich beetroot juice (∼400 mg nitrate) and were still elevated 4 weeks after daily consumption of 2 × 70 mL of this juice (∼800 mg nitrate per day), but not the placebo (Fig. 3). Plasma and salivary nitrite levels also increased 3 hours following the first dose and after the 4-week intervention with regular intake of nitrate-rich beetroot juice. The observed increases in plasma nitrate and nitrite suggest a functioning conversion of nitrate to nitrite through the nitrate-nitrite-NO pathway in the participants of this study, and that they were exposed to increases in nitrate and its metabolites following increased nitrate intake.

Nitrate and nitrite remained increased to more than 4-fold levels in plasma and to more than 3-fold levels in saliva after the 4-week nitrate intervention period (relative to baseline), even though participants fasted overnight and consumed their final juice beverage ≥12 hours before their study visit at the end of this period (Fig. 3). Circulating nitrate and nitrite depend on when nitrate is last ingested.^20^ Findings from previous studies indicated that plasma nitrate and nitrite increased in a dose-dependent manner,^36^ reached peak levels between 2–3 hours,^36,37^ remained elevated until 12 hours,^36^ and returned to baseline 24 hours after the ingestion of single doses of nitrate-rich beetroot juice in healthy individuals.^37^ Data suggest that the half-life of nitrate in plasma is 5–6 hours.^38^ Whether endogenous nitrate reservoirs might affect nitrate and nitrite in the circulation is largely unknown.^33,36^ Findings from previous clinical studies examining the effects of dietary nitrate intake over ≥4 weeks in middle-aged and older adults with above–normal blood pressure also showed increases in nitrate and nitrite in plasma and saliva following overnight fasting at the end of the intervention periods, although to varying extents.^16,20,21^ Compared to data from some other studies,^20,21^ nitrate and nitrite levels 4 weeks after the nitrate-based intervention in the current study were relatively high. Possible factors that may explain variations in these findings include inter-individual variability in pharmacodynamics, and differences in dose, form, and timing of nitrate intake.^20,36^ In our study, the daily consumption of 2 × ∼400 mg nitrate in the easily absorbable form of concentrated beetroot juice provided a constant and relatively high dose of nitrate, which may have contributed to the observed higher nitrate and nitrite levels. While the increase in nitrate and nitrite in plasma and saliva increased the potential for an augmented NO formation, this did not translate into sustained clinical effects, as discussed below.

### No sustained effects of dietary nitrate on vascular function or blood pressure

The development of vascular dysfunction is an important CVD risk factor especially with advancing age.^3,13^ Potential mechanisms by which vegetables, a central food group of cardiovascular health-promoting dietary patterns, elicit vascular benefits include the attenuation of vascular oxidative stress and inflammation and the improvement of NO bioavailability.^3,7,13^ Inorganic nitrate, through its effects on NO, appears to be a key bioactive vegetable compound that modulates some of these benefits.^3,8,9^ Previous studies examining the effects of nitrate on vascular function in humans have predominantly measured FMD.^8,23^ This non-invasive approach assesses the ability of peripheral conduit arteries to dilate in response to a mechanical (*i.e.*, blood flow) stimulus primarily through the endothelium-dependent production and release of NO *via* eNOS.^3,5^ Improvements in FMD have been observed between 1.5 and 3 hours following single doses (ranging from ∼75 to ∼750 mg nitrate) and longer-term (28 to 42 days) intake of nitrate (with doses ranging from 375 to 577 mg per day) in human studies,^8^ including middle aged and older adults with hypertension^16^ or other CVD risk factors.^15^ Other studies reported no effects on FMD,^21,22,39^ including a larger controlled study that evaluated the outcome of a 5-week intervention with leafy green vegetables or potassium nitrate pills (each containing ∼300 mg nitrate per day) in 231 hypertensive patients with an age between 50 and 70 years.^21^

By measuring the FBF response to brachial artery-infusion of ACh and GTN, we were able to assess ‘endothelium-dependent’ and ‘endothelium-independent’ mechanisms underlying function of peripheral resistance vessels, *i.e.*, microvascular function.^5,24,26^ Only a very few previous human studies have measured vasodilatory responses to ACh and sodium nitroprusside (SNP, another NO donor) to assess effects of nitrate interventions on microvascular function.^22,40^ By using laser Doppler perfusion imaging instead of plethysmography (as in our study), no^22^ or favorable^40^ nitrate effects on endothelial-dependent and -independent vasodilatation were observed in individuals with type 2 diabetes^22^ and individuals with Raynaud's phenomenon (a medical condition characterized by episodes of vasoconstriction).^40^ Gaining insight into the overall health and function of resistance arteries is important, as microvascular dysfunction is an early marker in the development of CVD.^24^ Another advantage of this method is that it enables to assess local effects of interventions with vasoactive factors (such as phytochemicals) on peripheral resistance vessel function without eliciting systemic effects.^26^ Moreover, bilateral FBF measurements in response to vasoactive substances *versus* control conditions greatly enhance the reproducibility as compared with unilateral measurements.^26^

In the currently reported study, no effects of nitrate (or the placebo) were detected on the FBF_Ach_ or FBF_GTN_-AUC ratios after compared with before the treatments, as the primary endpoint measure regarding FBF (Fig. 4). Contrary to our hypothesis, the daily intake 2 × 70 mL of beetroot juice containing ∼800 mg nitrate per day did not improve the endothelium-dependent FBF response to chemical stimulation (*i.e.*, ACh) in middle-aged and older men and woman with hypertension. Nor did the nitrate-based intervention affect the endothelium-independent response in FBF. Potential factors that could influence whether or how efficient an increased nitrate intake improves vascular function include the dose of nitrate provided and the age and (vascular) health status of the participants.^8^ The efficiency of converting nitrate into nitrite and NO and the NO responsiveness of the vasculature may be diminished with aging.^41^ The findings of our study suggest an efficient conversion of nitrate into nitrite. However, it remains possible that the further reduction of nitrite to NO or the vascular NO responsiveness might have been attenuated or that nitrate-derived NO might have interfered with eNOS activity (discussed below). Another possibility for the variance in the findings between previous studies and our investigation is that resistance compared to conduit arteries respond differentially to nitrate and NO. These aspects warrant further investigation.

Of note, compared to baseline, we observed within-group increases in the FBF_Ach_ and FBF_GTN_ responses calculated as AUC in the interventional *versus* control arm (as secondary endpoint measures) three hours following consumption of 70 mL of beetroot juice containing ∼400 mg nitrate (Fig. 5). Acute effects of nitrate on the vasculature have been mainly attributed to peripheral vasodilation mediated by NO.^11,37^ As an additional possible mechanism underlying the vasodilatory effects of nitrate, the generation of NO through the nitrate-nitrite-NO pathway may attenuate vascular oxidative stress by scavenging superoxide anions (O_2_^−^) and by modulating the activity of reactive oxygen species (ROS)-generating enzymes in the vasculature.^11,42^ Nitric oxide is a free radical, which is central to the mechanisms that underly physiological NO signaling.^9,11^ Nevertheless, NO is less reactive than other radicals and can also act as an antioxidant by scavenging more reactive radicals.^11^ Thereby, the nitrate-derived NO might improve the vascular ability of eNOS-derived NO and endothelium-dependent relaxation, and consequently, increase endothelium-dependent dilatation and blood blow, as assessed by FBF_ACh_. Such effects on the NO bioavailability may also explain the enhanced responsiveness to exogenous NO donors such as GTN or SNP. Additional research is required to verify whether our observations point toward subtle acute effects of nitrate on vascular function, and whether even smaller periodic improvements in vascular function could be clinically relevant.

Hypertension is another main CVD risk factor, and there is evidence that high blood pressure contributes to the greater absolute risk of incident CVD in older compared with younger adults.^4^ With regards to dietary interventions to prevent or treat hypertension, inorganic nitrate has been identified as a plant compound that may importantly contribute to the blood pressure-lowering effects of diets rich in plant foods, such as the DASH (Dietary Approaches to Stop Hypertension) diet.^8,35^ The reduction of blood pressure in normotensive and hypertensive individuals is the most consistent outcome of increased dietary nitrate consumption in human studies.^8,11,23,37^ Acute blood pressure-lowering effects of nitrate have been reported in most previous studies investigating blood pressure responses to single nitrate doses (ranging from 182 to 1488 mg nitrate) between 2 and 24 hours after ingestion.^8,23^ However, findings on the longer-term effects on blood pressure are mixed.^8,23^ A *meta*-analysis of 23 clinical studies conducted prior to 2018 indicated a clear benefit of nitrate on blood pressure, but this analysis also suggested that the nitrate apparently has a greater effect on lowering resting (clinic) blood pressure than 24-hour ambulatory blood pressure.^23^

In the present study, clinic systolic and mean arterial blood pressure decreased 3 hours following the intake of the first dose of beetroot juice containing ∼400 mg nitrate, but not the placebo (Table 2). Contrary to the acute responses, the daily consumption of ∼800 mg nitrate through beetroot juice over 4 weeks did not sustainably lower blood pressure (Table 2). Instead, a reduction in clinic systolic and mean arterial blood pressure was observed following the daily intake of nitrate-depleted beetroot juice. It may be speculated that bioactive compounds other than nitrate contained in the beetroot juice, including betalains and flavonoids, may have affected blood pressure.^43^ Nevertheless, while standard procedures were used for measuring blood pressure at our clinic, data obtained from clinic blood pressure measurements should be interpreted with caution as individual blood pressure measurements tend to vary in an unpredictable and random fashion.^4^ To provide more reliable blood pressure measures, the outcome of the 4-week treatment periods was therefore also assessed by home and 24-hour ambulatory blood pressure measurements. Twenty four-hour ambulatory blood pressure monitoring is considered the ‘gold standard’ measurement of blood pressure.^4^ In other studies that monitored 24-hour ambulatory blood pressure following regular nitrate consumption over a period between 7 and 35 days, with daily doses ranging between 165 and 600 mg,^8,21,23^ either reductions^16^ or no changes in blood pressure^20,21,44^ were observed in various populations, including hypertensive middle-aged and older adults.^16,20,21^

The findings of the present study agree with several previous investigations that reported a lack of longer-term blood pressure-lowering effects with home- and 24-hour ambulatory blood pressure measurements.^20,21^ A possible mechanism for why nitrate-based interventions do not consistently evoke sustained effects on vascular function and blood pressure could be an interaction of the dietary nitrate-nitrite-NO pathway with the endogenous eNOS-dependent pathway.^45^ The data from the current study do not enable firm conclusions about possible interactive effects of nitrate-derived NO with eNOS activity and, specifically, about whether the daily dose of ∼800 mg nitrate with regular beetroot juice consumption was too high. However, previous studies in animal models reported that very high doses of nitrate from drinking water had no effect^46^ or even attenuated^45^ ACh-mediated vasorelaxation. This supports the notion that NO derived from chronic higher-dosed nitrate intake might interfere with endogenous vascular NO generation, possibly through negative feedback mechanisms affecting eNOS expression and/or eNOS activity.^45^ Additional research is required to investigate whether there is a dose range in which nitrate from plant-derived sources might exert more pronounced or consistent physiological and clinical effects.

## Conclusion

There are several aspects that warrant consideration from theoretical and practical viewpoints. Recognizing the individual variation in some of our data, we might have been able to detect subtle differences between the groups with a larger sample size. Furthermore, acknowledging emerging data suggesting differential responses to nitrate in men as compared with women,^47,48^ a larger number of both male and female study participants may have enabled us to assess if there is any evidence of sex-related effects. Considering that we pre-screened 409 volunteers and that 389 of them were excluded during the screening procedures, mainly due to use of medications specified as exclusion criteria, this made recruiting eligible participants difficult. Stringent exclusion criteria were used regarding concomitant medication use to avoid interference of drugs with nitrate. However, this approach also emphasizes the value of this study with regards to its internal validity. In particular, we took thorough measures to control factors known to affect nitrate metabolism and bioactivation (including use of NO donors, organic nitrates/nitrites, sildenafil and related drugs, antibacterial mouthwash, antibiotics, as well as the background diet). Based on estimations from the food frequency questionnaire data (obtained at all four study days) and available databases,^49,50^ the nitrate intake from the background diet during the study was low (*i.e.*, 40 ± 12 mg per day, values are mean ± SD; unpublished findings).

Further strengths of this clinical study include its strong experimental design, the use of FBF measurements in response to ACh and GTN, as well as 24-hour ambulatory blood pressure monitoring combined with home blood pressure measurements. These methods are widely considered as ‘gold standard’-approaches for assessing vascular function and blood pressure. From a practical viewpoint, the results of our study suggest no long-term effects on vascular function or blood pressure from the daily consumption of 2 × ∼400 mg of inorganic nitrate through beetroot juice in middle-aged and older men and women with treated hypertension. Further investigations are needed to examine why the increased plasma and salivary nitrate and nitrite levels following the 4-week nitrate intervention period did not translate into sustained clinical benefits. Future studies may focus on whether there are thresholds in nitrate doses, below or above which inorganic nitrate from vegetable sources is less effective. Together, the findings from this study provide new information on the efficacy of vegetable juice-derived nitrate as a nutritional adjuvant to improve vascular function, treat elevated blood pressure, and counteract vascular aging in an older population at a heightened CVD risk. These data form an important basis for further translational research aimed at ‘refining’ such dietary interventions.

## Author contributions

Conceptualization: NP, K-HW, JMP, RJW, CPB, JMH, MW, ON. Data curation: RF, ON. Formal analysis: RF, ML, SW. Funding acquisition: ON. Investigation: RF, ML, SW. Methodology: ML, SW, H-PS, RJW, KDC, MW, ON. Project administration: RF, MW, ON. Resources: K-HW, KDC, MW, ON. Software: RF, NP, ML. Supervision: K-HW, MW, ON. Validation: ON. Visualization: RF, ML. Writing – original draft: RF, ON. Writing – review & editing: RF, ML, SW, NP, K-HW, H-PS, JMP, CPB, JMH, MW, ON.

## Conflicts of interest

There are no conflicts to declare.

## Supplementary Material

FO-015-D3FO03749E-s001

FO-015-D3FO03749E-s002

FO-015-D3FO03749E-s003

## References

[cit1] Tsao C. W., Aday A. W., Almarzooq Z. I., Alonso A., Beaton A. Z., Bittencourt M. S., Boehme A. K., Buxton A. E., Carson A. P., Commodore-Mensah Y., Elkind M. S. V., Evenson K. R., Eze-Nliam C., Ferguson J. F., Generoso G., Ho J. E., Kalani R., Khan S. S., Kissela B. M., Knutson K. L., Levine D. A., Lewis T. T., Liu J., Loop M. S., Ma J., Mussolino M. E., Navaneethan S. D., Perak A. M., Poudel R., Rezk-Hanna M., Roth G. A., Schroeder E. B., Shah S. H., Thacker E. L., VanWagner L. B., Virani S. S., Voecks J. H., Wang N. Y., Yaffe K., Martin S. S. (2022). Heart Disease and Stroke Statistics-2022 Update: A Report From the American Heart Association. Circulation.

[cit2] Timmis A., Townsend N., Gale C. P., Torbica A., Lettino M., Petersen S. E., Mossialos E. A., Maggioni A. P., Kazakiewicz D., May H. T., De Smedt D., Flather M., Zuhlke L., Beltrame J. F., Huculeci R., Tavazzi L., Hindricks G., Bax J., Casadei B., Achenbach S., Wright L., Vardas P., European Society of Cardiology (2020). European Society of Cardiology: Cardiovascular Disease Statistics 2019. Eur. Heart J..

[cit3] Rossman M. J., LaRocca T. J., Martens C. R., Seals D. R. (2018). Healthy lifestyle-based approaches for successful vascular aging. J. Appl. Physiol..

[cit4] Whelton P. K., Carey R. M., Aronow W. S., Casey Jr. D. E., Collins K. J., Dennison Himmelfarb C., DePalma S. M., Gidding S., Jamerson K. A., Jones D. W., MacLaughlin E. J., Muntner P., Ovbiagele B., Smith Jr. S. C., Spencer C. C., Stafford R. S., Taler S. J., Thomas R. J., Williams Sr. K. A., Williamson J. D., Wright Jr. J. T. (2018). 2017 ACC/AHA/AAPA/ABC/ACPM/AGS/APhA/ASH/ASPC/NMA/PCNA Guideline for the Prevention, Detection, Evaluation, and Management of High Blood Pressure in Adults: Executive Summary: A Report of the American College of Cardiology/American Heart Association Task Force on Clinical Practice Guidelines. Circulation.

[cit5] Seals D. R., Jablonski K. L., Donato A. J. (2011). Aging and vascular endothelial function in humans. Clin. Sci..

[cit6] Lichtenstein A. H., Appel L. J., Vadiveloo M., Hu F. B., Kris-Etherton P. M., Rebholz C. M., Sacks F. M., Thorndike A. N., Van Horn L., Wylie-Rosett J. (2021). 2021 Dietary Guidance to Improve Cardiovascular Health: A Scientific Statement From the American Heart Association. Circulation.

[cit7] Aune D., Giovannucci E., Boffetta P., Fadnes L. T., Keum N., Norat T., Greenwood D. C., Riboli E., Vatten L. J., Tonstad S. (2017). Fruit and vegetable intake and the risk of cardiovascular disease, total cancer and all-cause mortality-a systematic review and dose-response meta-analysis of prospective studies. Int. J. Epidemiol..

[cit8] Blekkenhorst L. C., Bondonno N. P., Liu A. H., Ward N. C., Prince R. L., Lewis J. R., Devine A., Croft K. D., Hodgson J. M., Bondonno C. P. (2018). Nitrate, the oral microbiome, and cardiovascular health: a systematic literature review of human and animal studies. Am. J. Clin. Nutr..

[cit9] Raubenheimer K., Bondonno C., Blekkenhorst L., Wagner K. H., Peake J. M., Neubauer O. (2019). Effects of dietary nitrate on inflammation and immune function, and implications for cardiovascular health. Nutr. Rev..

[cit10] Woessner M. N., McIlvenna L. C., Ortiz de Zevallos J., Neil C. J., Allen J. D. (2018). Dietary nitrate supplementation in cardiovascular health: an ergogenic aid or exercise therapeutic?. Am. J. Physiol.: Heart Circ. Physiol..

[cit11] Carlstrom M., Lundberg J. O., Weitzberg E. (2018). Mechanisms underlying blood pressure reduction by dietary inorganic nitrate. Acta Physiol..

[cit12] Lundberg J. O., Gladwin M. T., Weitzberg E. (2015). Strategies to increase nitric oxide signalling in cardiovascular disease. Nat. Rev. Drug Discovery.

[cit13] Seals D. R., Kaplon R. E., Gioscia-Ryan R. A., LaRocca T. J. (2014). You're only as old as your arteries: translational strategies for preserving vascular endothelial function with aging. Physiology.

[cit14] Casey D. P., Bock J. M. (2021). Inorganic nitrate supplementation attenuates conduit artery retrograde and oscillatory shear in older adults. Am. J. Physiol.: Heart Circ. Physiol..

[cit15] Velmurugan S., Gan J. M., Rathod K. S., Khambata R. S., Ghosh S. M., Hartley A., Van Eijl S., Sagi-Kiss V., Chowdhury T. A., Curtis M., Kuhnle G. G., Wade W. G., Ahluwalia A. (2016). Dietary nitrate improves vascular function in patients with hypercholesterolemia: a randomized, double-blind, placebo-controlled study. Am. J. Clin. Nutr..

[cit16] Kapil V., Khambata R. S., Robertson A., Caulfield M. J., Ahluwalia A. (2015). Dietary nitrate provides sustained blood pressure lowering in hypertensive patients: a randomized, phase 2, double-blind, placebo-controlled study. Hypertension.

[cit17] Bondonno C. P., Yang X., Croft K. D., Considine M. J., Ward N. C., Rich L., Puddey I. B., Swinny E., Mubarak A., Hodgson J. M. (2012). Flavonoid-rich apples and nitrate-rich spinach augment nitric oxide status and improve endothelial function in healthy men and women: a randomized controlled trial. Free Radicals Biol. Med..

[cit18] Broxterman R. M., La Salle D. T., Zhao J., Reese V. R., Richardson R. S., Trinity J. D. (2019). Influence of dietary inorganic nitrate on blood pressure and vascular function in hypertension: prospective implications for adjunctive treatment. J. Appl. Physiol..

[cit19] Raubenheimer K., Hickey D., Leveritt M., Fassett R., Ortiz de Zevallos Munoz J., Allen J. D., Briskey D., Parker T. J., Kerr G., Peake J. M., Pecheniuk N. M., Neubauer O. (2017). Acute Effects of Nitrate-Rich Beetroot Juice on Blood Pressure, Hemostasis and Vascular Inflammation Markers in Healthy Older Adults: A Randomized, Placebo-Controlled Crossover Study. Nutrients.

[cit20] Blekkenhorst L. C., Lewis J. R., Prince R. L., Devine A., Bondonno N. P., Bondonno C. P., Wood L. G., Puddey I. B., Ward N. C., Croft K. D., Woodman R. J., Beilin L. J., Hodgson J. M. (2018). Nitrate-rich vegetables do not lower blood pressure in individuals with mildly elevated blood pressure: a 4-wk randomized controlled crossover trial. Am. J. Clin. Nutr..

[cit21] Sundqvist M. L., Larsen F. J., Carlstrom M., Bottai M., Pernow J., Hellenius M. L., Weitzberg E., Lundberg J. O. (2020). A randomized clinical trial of the effects of leafy green vegetables and inorganic nitrate on blood pressure. Am. J. Clin. Nutr..

[cit22] Gilchrist M., Winyard P. G., Aizawa K., Anning C., Shore A., Benjamin N. (2013). Effect of dietary nitrate on blood pressure, endothelial function, and insulin sensitivity in type 2 diabetes. Free Radicals Biol. Med..

[cit23] Jackson J. K., Patterson A. J., MacDonald-Wicks L. K., Oldmeadow C., McEvoy M. A. (2018). The role of inorganic nitrate and nitrite in cardiovascular disease risk factors: a systematic review and meta-analysis of human evidence. Nutr. Rev..

[cit24] Masi S., Rizzoni D., Taddei S., Widmer R. J., Montezano A. C., Luscher T. F., Schiffrin E. L., Touyz R. M., Paneni F., Lerman A., Lanza G. A., Virdis A. (2021). Assessment and pathophysiology of microvascular disease: recent progress and clinical implications. Eur. Heart J..

[cit25] Gutterman D. D., Chabowski D. S., Kadlec A. O., Durand M. J., Freed J. K., Ait-Aissa K., Beyer A. M. (2016). The Human Microcirculation: Regulation of Flow and Beyond. Circ. Res..

[cit26] Wilkinson I. B., Webb D. J. (2001). Venous occlusion plethysmography in cardiovascular research: methodology and clinical applications. Br. J. Clin. Pharmacol..

[cit27] Petrie J. R., Ueda S., Morris A. D., Murray L. S., Elliott H. L., Connell J. M. (1998). How reproducible is bilateral forearm plethysmography?. Br. J. Clin. Pharmacol..

[cit28] Weisshaar S., Litschauer B., Eipeldauer M., Hobl E. L., Wolzt M. (2017). Ticagrelor mitigates ischaemia-reperfusion induced vascular endothelial dysfunction in healthy young males - a randomized, single-blinded study. Br. J. Clin. Pharmacol..

[cit29] Lind L., Berglund L., Larsson A., Sundstrom J. (2011). Endothelial function in resistance and conduit arteries and 5-year risk of cardiovascular disease. Circulation.

[cit30] Lakatta E. G., Levy D. (2003). Arterial and cardiac aging: major shareholders in cardiovascular disease enterprises: Part I: aging arteries: a “set up” for vascular disease. Circulation.

[cit31] Fuchs R., Klaperski S., Gerber M., Seelig H. (2015). Measurement of physical activity and sport activity with the BSA questionnaire [Messung der Bewegungs- und Sportaktivität mit dem BSA-Fragebogen]. Zeitschrift für Gesundheitspsychologie.

[cit32] Haftenberger M., Heuer T., Heidemann C., Kube F., Krems C., Mensink G. B. (2010). Relative validation of a food frequency questionnaire for national health and nutrition monitoring. Nutr. J..

[cit33] Jones A. M., Vanhatalo A., Seals D. R., Rossman M. J., Piknova B., Jonvik K. L. (2021). Dietary Nitrate and Nitric Oxide Metabolism: Mouth, Circulation, Skeletal Muscle, and Exercise Performance. Med. Sci. Sports Exercise.

[cit34] Yang X., Bondonno C. P., Indrawan A., Hodgson J. M., Croft K. D. (2013). An improved mass spectrometry-based measurement of NO metabolites in biological fluids. Free Radicals Biol. Med..

[cit35] Bondonno C. P., Zhong L., Bondonno N. P., Sim M., Blekkenhorst L. C., Liu A., Rajendra A., Pokharel P., Erichsen D. W., Neubauer O., Croft K. D., Hodgson J. M. (2023). Nitrate: The Dr. Jekyll and Mr. Hyde of human health?. Trends Food Sci. Technol..

[cit36] Wylie L. J., Kelly J., Bailey S. J., Blackwell J. R., Skiba P. F., Winyard P. G., Jeukendrup A. E., Vanhatalo A., Jones A. M. (2013). Beetroot juice and exercise: pharmacodynamic and dose-response relationships. J. Appl. Physiol..

[cit37] Webb A. J., Patel N., Loukogeorgakis S., Okorie M., Aboud Z., Misra S., Rashid R., Miall P., Deanfield J., Benjamin N., MacAllister R., Hobbs A. J., Ahluwalia A. (2008). Acute blood pressure lowering, vasoprotective, and antiplatelet properties of dietary nitrate via bioconversion to nitrite. Hypertension.

[cit38] Omar S. A., Webb A. J., Lundberg J. O., Weitzberg E. (2016). Therapeutic effects of inorganic nitrate and nitrite in cardiovascular and metabolic diseases. J. Intern. Med..

[cit39] Bahra M., Kapil V., Pearl V., Ghosh S., Ahluwalia A. (2012). Inorganic nitrate ingestion improves vascular compliance but does not alter flow-mediated dilatation in healthy volunteers. Nitric Oxide.

[cit40] Shepherd A. I., Costello J. T., Bailey S. J., Bishop N., Wadley A. J., Young-Min S., Gilchrist M., Mayes H., White D., Gorczynski P., Saynor Z. L., Massey H., Eglin C. M. (2019). “Beet” the cold: beetroot juice supplementation improves peripheral blood flow, endothelial function, and anti-inflammatory status in individuals with Raynaud's phenomenon. J. Appl. Physiol..

[cit41] Lara J., Ashor A. W., Oggioni C., Ahluwalia A., Mathers J. C., Siervo M. (2016). Effects of inorganic nitrate and beetroot supplementation on endothelial function: a systematic review and meta-analysis. Eur. J. Nutr..

[cit42] Carlstrom M., Persson A. E., Larsson E., Hezel M., Scheffer P. G., Teerlink T., Weitzberg E., Lundberg J. O. (2011). Dietary nitrate attenuates oxidative stress, prevents cardiac and renal injuries, and reduces blood pressure in salt-induced hypertension. Cardiovasc. Res..

[cit43] Parmenter B. H., Croft K. D., Cribb L., Cooke M. B., Bondonno C. P., Lea A., McPhee G. M., Komanduri M., Nolidin K., Savage K., Pase M. P., Hodgson J. M., Stough C., Bondonno N. P. (2021). Higher habitual dietary flavonoid intake associates with lower central blood pressure and arterial stiffness in healthy older adults. Br. J. Nutr..

[cit44] Jajja A., Sutyarjoko A., Lara J., Rennie K., Brandt K., Qadir O., Siervo M. (2014). Beetroot supplementation lowers daily systolic blood pressure in older, overweight subjects. Nutr. Res..

[cit45] Carlstrom M., Liu M., Yang T., Zollbrecht C., Huang L., Peleli M., Borniquel S., Kishikawa H., Hezel M., Persson A. E., Weitzberg E., Lundberg J. O. (2015). Cross-talk Between Nitrate-Nitrite-NO and NO Synthase Pathways in Control of Vascular NO Homeostasis. Antioxid. Redox Signal..

[cit46] Bakker J. R., Bondonno N. P., Gaspari T. A., Kemp-Harper B. K., McCashney A. J., Hodgson J. M., Croft K. D., Ward N. C. (2016). Low dose dietary nitrate improves endothelial dysfunction and plaque stability in the ApoE(-/-) mouse fed a high fat diet. Free Radicals Biol. Med..

[cit47] Baranauskas M. N., Freemas J. A., Tan R., Carter S. J. (2022). Moving beyond inclusion: Methodological considerations
for the menstrual cycle and menopause in research evaluating effects of dietary nitrate on vascular function. Nitric Oxide.

[cit48] Ortiz de Zevallos J., Hogwood A. C., Kruse K., De Guzman J., Buckley M., Weltman A. L., Allen J. D. (2023). Sex differences in the effects of inorganic nitrate supplementation on exercise economy and endurance capacity in healthy young adults. J. Appl. Physiol..

[cit49] Zhong L., Blekkenhorst L. C., Bondonno N. P., Sim M., Woodman R. J., Croft K. D., Lewis J. R., Hodgson J. M., Bondonno C. P. (2022). A food composition database for assessing nitrate intake from plant-based foods. Food Chem..

[cit50] Blekkenhorst L. C., Prince R. L., Ward N. C., Croft K. D., Lewis J. R., Devine A., Shinde S., Woodman R. J., Hodgson J. M., Bondonno C. P. (2017). Development of a reference database for assessing dietary nitrate in vegetables. Mol. Nutr. Food Res..

