# Supplementary figures and images for: Increased nitrate intake from beetroot juice over 4 weeks affects nitrate metabolism, but not vascular function or blood pressure in older adults with hypertension
Source: Food Funct. 2024 Mar 25;15(8):4065–78. doi: 10.1039/d3fo03749e (PMC11034575; doi:10.1039/d3fo03749e)

## Acetylcholine [nmol\*1/min]

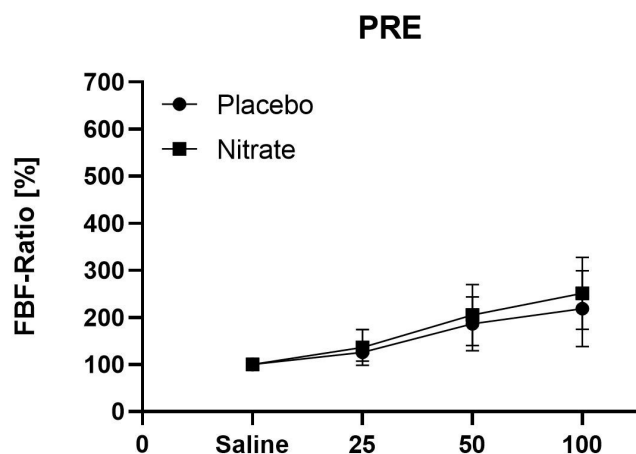

## Glyceryltrinitrate [nmol\*1/min]

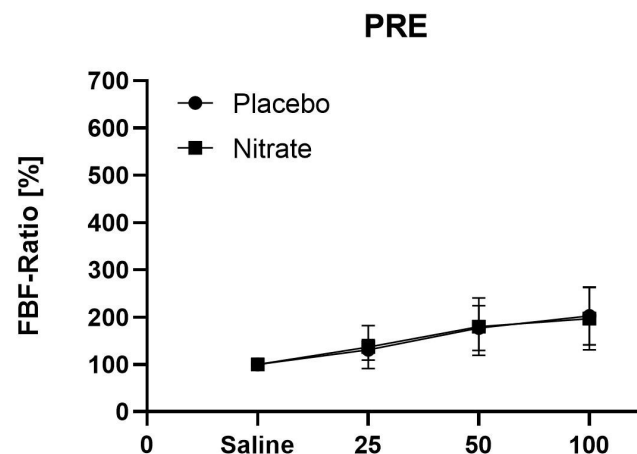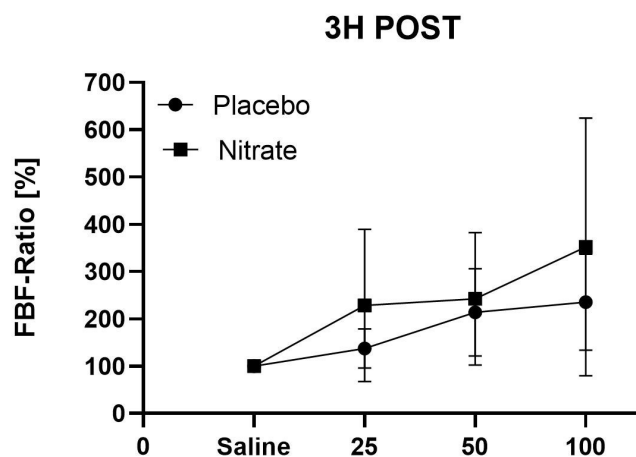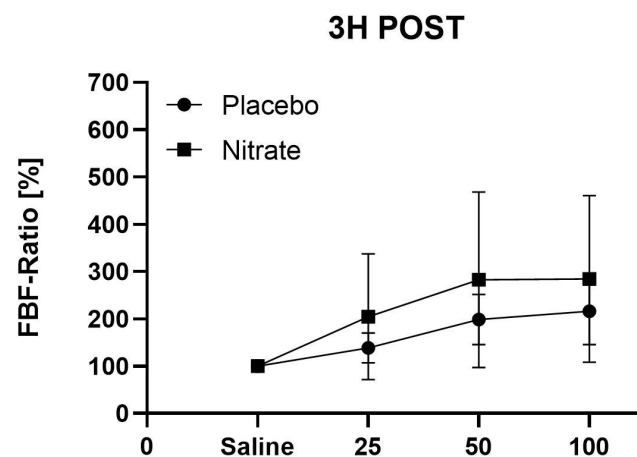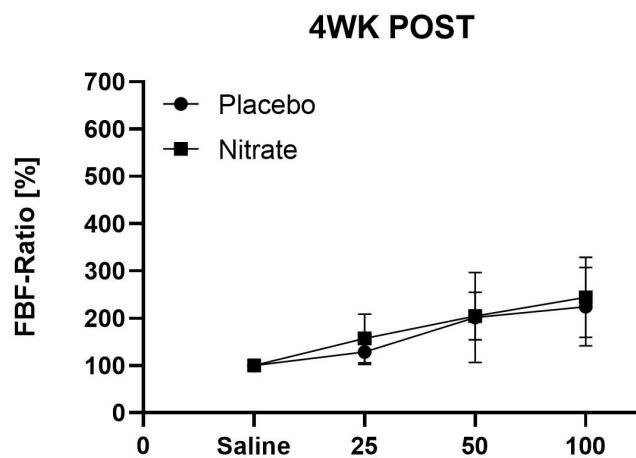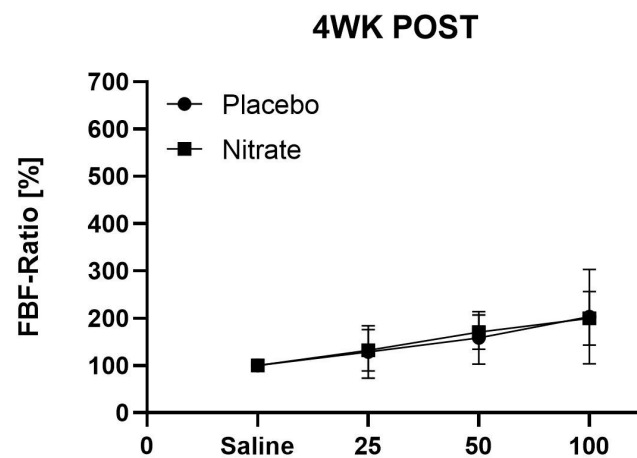

Supplement: FO-015-D3FO03749E-s002 [file FO-015-D3FO03749E-s002.pdf]
