# Supplementary material for: Increased nitrate intake from beetroot juice over 4 weeks affects nitrate metabolism, but not vascular function or blood pressure in older adults with hypertension
Source: Food Funct. 2024 Mar 25;15(8):4065–78. doi: 10.1039/d3fo03749e (PMC11034575; doi:10.1039/d3fo03749e)
Supplement: FO-015-D3FO03749E-s003 [file FO-015-D3FO03749E-s003.pdf]

Electronic Supplementary Material (ESI) for Food & Function.
This journal is © The Royal Society of Chemistry 2024

Supplemental Table 1. Absolute forearm blood flow (FBF) ($ml \cdot min^{-1} \cdot 100\ ml^{-1}$) of the interventional- and control arm during intra-arterial saline infusion before (PRE), and 3 hours (3H POST) and 4 weeks (4WK POST) after the interventions with nitrate-rich (Nitrate) or nitrate-depleted (Placebo) beetroot juice.

	**Nitrate**			
	Basal FBF prior to acetylcholine infusion		Basal FBF prior to glyceryltrinitrate infusion	
	Interventional arm	Control arm	Interventional arm	Control arm
PRE	5.7 ± 1.1	6.1 ± 1.2	5.6 ± 1.4	5.8 ± 1.9
3H POST	4.4 ± 1.2	4.9 ± 1.1	4.3 ± 1.2	4.6 ± 1.0
4WK POST	6.1 ± 1.0	7.3 ± 1.7	5.5 ± 1.1	5.9 ± 1.6
	**Placebo**			
	Basal FBF prior to acetylcholine infusion		Basal FBF prior to glyceryltrinitrate infusion	
	Interventional arm	Control arm	Interventional arm	Control arm
PRE	5.9 ± 1.1	6.3 ± 1.3	5.7 ± 1.2	6.3 ± 1.3
3H POST	5.1 ± 0.9	5.6 ± 1.1	5.1 ± 1.2	5.4 ± 1.0
4WK POST	5.9 ± 1.2	7.2 ± 1.9	5.8 ± 1.1	6.9 ± 2.3

Values are mean ± 95% confidence interval. Data were compared using a repeated-measures linear mixed-model.
